# Rebuilding the Missing Part—A Review on Photoreceptor Transplantation

**DOI:** 10.3389/fnsys.2016.00105

**Published:** 2017-01-05

**Authors:** Tiago F. Santos-Ferreira, Oliver Borsch, Marius Ader

**Affiliations:** DFG-Center for Regenerative Therapies Dresden, Cluster of Excellence, Technische Universität DresdenDresden, Germany

**Keywords:** retina, photoreceptor, retinal degeneration, transplantation, embryonic stem cell, induced pluripotent stem cell, pre-clinical mouse models, cytoplasmic exchange

## Abstract

Vision represents one of the main senses for humans to interact with their environment. Our sight relies on the presence of fully functional light sensitive cells – rod and cone photoreceptors — allowing us to see under dim (rods) and bright (cones) light conditions. Photoreceptor degeneration is one of the major causes for vision impairment in industrialized countries and it is highly predominant in the population above the age of 50. Thus, with the continuous increase in life expectancy it will make retinal degeneration reach an epidemic proportion. To date, there is no cure established for photoreceptor loss, but several therapeutic approaches, spanning from neuroprotection, pharmacological drugs, gene therapy, retinal prosthesis, and cell (RPE or photoreceptor) transplantation, have been developed over the last decade with some already introduced in clinical trials. In this review, we focus on current developments in photoreceptor transplantation strategies, its major breakthroughs, current limitations and the next challenges to translate such cell-based approaches toward clinical application.

## Introduction

The ability to visually perceive the surrounding world is provided by an elegantly designed organ—the eye—which contains light sensing cells: rod and cone photoreceptors. Several diseases, such as retinitis pigmentosa (RP, Hartong et al., 2006), Leber's Congenital Amaurosis (LCA, den Hollander et al., 2008) and age-related macular degeneration (AMD, Jager et al., 2008) result in the functional impairment and loss of photoreceptors leading to vision impairment and ultimately blindness. Blindness alone affects 34 million people worldwide and, with an ever-increasing population and its continuous aging, blinding diseases may reach epidemic levels (WHO, 2016, Visual impairment and blindness). The perspective of an even “darker” future has led to the development of multiple therapeutic approaches including neuroprotection (Trifunović et al., 2012), gene therapy (Acland et al., 2001), antibody therapy (Lazic and Gabric, 2007), optogenetics (Busskamp et al., 2010), retinal prosthesis (Lewis et al., 2016), and cell replacement therapy.

**Figure 1:**
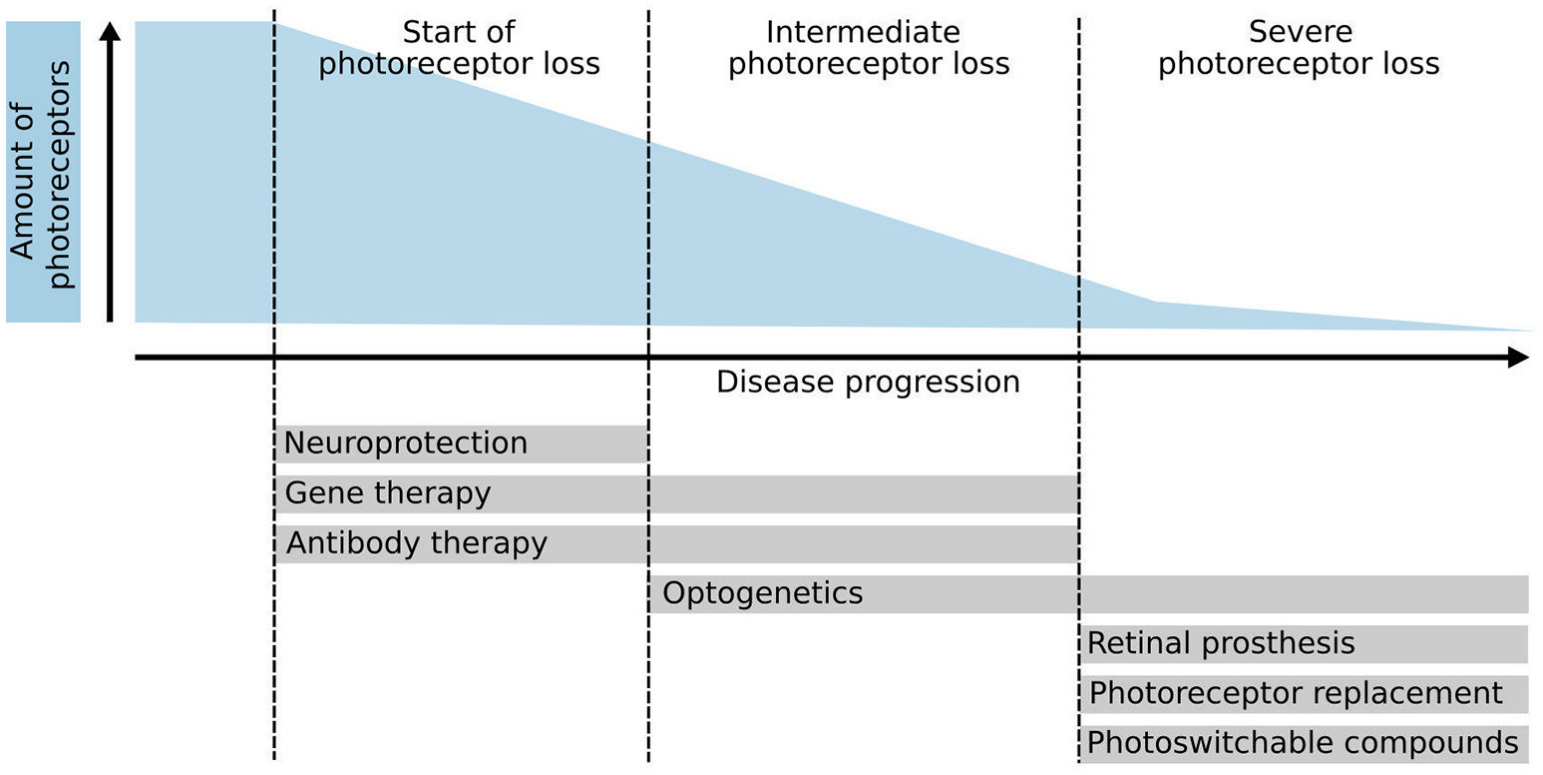
**Photoreceptor degeneration and potential therapies**. Schematic overview of several therapeutic approaches and their application based on the disease progression and amount of photoreceptors left. Initial therapeutic approaches such as neuroprotection, gene therapy, and antibody therapy require the presence of endogenous photoreceptors to be effective. Conversely, optogenetics, retinal prosthesis, photoreceptor replacement therapies, and photo-switchable compounds aim to treat patients in the late stages of the disease where few or no photoreceptors are left. Optogenetic approaches can also be used to reactivate remaining but dysfunctional cone photoreceptors.

Whereas, some of these treatment approaches aim to rescue remaining photoreceptors and thus have to be applied at early stages of the disease (e.g., gene therapy, neuroprotection, pharmacology), other therapeutic interventions target late disease stages, when the majority or all photoreceptors have been lost, making them particularly challenging (Figure 1). Interestingly, the neurons of the inner nuclear layer (INL), i.e., horizontals, bipolars, and amacrines, survive for extended time periods after photoreceptor degeneration despite significant dendritic retraction and circuit remodeling. Therefore, one strategy is to prevent degeneration of second-order neurons combined with the restoration of photoreceptor-mediated light sensitivity in order to take advantage of the processing within the natural retinal circuitry (Jones et al., 2016; Reh, 2016). To bypass the loss of sensory input, optogenetic tools, or photoswitchable compounds (Tochitsky et al., 2016) have been explored, which either make bipolar or ganglion cells light-sensitive or even re-activate remaining cone photoreceptors (Busskamp et al., 2010) in diseases such as retinitis pigmentosa. Moreover, recently the grafting of artificial light sensing devices, i.e., retinal prostheses, that provide INL neurons with electrical stimuli, received FDA approval and have restored some basic visual function to affected patients in first clinical applications (da Cruz et al., 2016).

Thus, diseases characterized by photoreceptor loss might be also amenable to cell replacement therapies based on the following reasons: first, the eye is a separated organ that can be relatively easily accessed and manipulated. Second, photoreceptors are located adjacent to an epithelial layer—the retinal-pigmented epithelium (RPE). The contact between RPE and the outer segments (OSs) of photoreceptors is relative loose and creates a natural cleft that can be separated upon injection of a solution containing donor cells. Third, the eye represents a partially immune-privileged organ that is less prone to immune rejection of foreign material (Streilein et al., 2002; Forrester, 2008), and fourth, transplanted photoreceptors have to extend their axons just a few micrometers in order to establish synaptic connections to horizontal and bipolar cells.

In this review, we will provide an overview of photoreceptor transplantation studies and in which directions the field is currently moving. Additionally, we will refer and discuss a critical concept: structural integration of donor cells into the host retinal tissue. Until very recently donor photoreceptors were understood to physically migrate and integrate into the recipient outer nuclear layer (ONL) as identified by fluorescent donor proteins being present within the host tissue. However, three recent studies provided evidence that structural integration represents a minor mechanism for this observation and that exchange of cell material between donor and host photoreceptors account for the majority of reporter labeled cells seen in the host retina (Pearson et al., 2016; Santos-Ferreira et al., 2016a; Singh et al., 2016). These novel results are discussed in detail in section “Paradigm Shift in Photoreceptor Replacement Therapy”, but for simplicity purposes and taking in account the historical context, we will describe all reporter labeled cells found within the host retina of previous studies as “integrated” or refer generally to improved/reduced transplantation outcomes.

## Development of photoreceptor replacement therapy

In the late 80s Del Cerro, Silvermann, Hughes, and Gouras pioneered photoreceptor transplantation into the subretinal space of rats or mice. Despite technical limitations at the time, key ideas such as the need of tracing grafted cells (del Cerro et al., 1989a,b; Gouras et al., 1991a,b,c; Du et al., 1992) or enriching donor cells (by ONL vibratome sectioning or micro-aggregates; Gouras et al., 1994) prior to transplantation, were already explored. The potential of photoreceptor replacement was evident in these initial studies that showed graft-survival up to 9 months in wild-type hosts or in a mouse model of retina degeneration, formation of primitive outer-segments (OS) and synapses (del Cerro et al., 1989a; Gouras et al., 1991a,b,c, 1994; Du et al., 1992).

Several studies then aimed for the replacement of lost photoreceptors using expanded retinal progenitor cells, primary retinal- or photoreceptor sheets (Coles et al., 2004; Klassen et al., 2004; Merhi-Soussi et al., 2006), however, full differentiation of donor cells into mature photoreceptor phenotypes and significant visual improvement was rarely observed arguing for further improvement of such approaches (Ballios et al., 2015). Particularly, the use of *in vitro* expanded retinal progenitor cells (RPCs), also termed retinal stem cells, for photoreceptor replacement (Klassen et al., 2004) has been recently challenged by studies that suggested limited differentiation potential of these cells toward the photoreceptor lineage (Cicero et al., 2009; Gualdoni et al., 2010; Czekaj et al., 2012; Ringuette et al., 2014). Nevertheless, recently the first clinical trial using *in vitro* expanded human RPCs has been initiated (jCYTE; www.clinicaltrials.gov; ID: NCT02320812).

## Young post-mitotic photoreceptors show improved transplantation outcome

A major breakthrough in photoreceptor transplantation was achieved by MacLaren et al. (2006) followed by Bartsch et al. (2008) wherein the optimal age of donor photoreceptors for transplantation was identified resulting in higher survival and integration rates. Young post-mitotic rod photoreceptors from eGFP expressing reporter mouse lines isolated using fluorescent activated cell sorting (FACS) at the peak of rod genesis, i.e., at postnatal day (P) 4–6, were shown to integrate more efficiently into wild-type and degenerating retinas than retinal cells isolated at earlier or later ontogenetic stages [i.e., from embryonic day (E) 11.5 to adult]. Following grafting, GFP^+^ cells had their cell bodies properly located within the host ONL and formed synaptic terminals and outer segments resembling a mature rod photoreceptor phenotype (MacLaren et al., 2006; Bartsch et al., 2008). Integrated donor cells generated the distinct photoreceptor synaptic triad connecting to horizontal and bipolar dendrites (Pearson et al., 2012) and formed outer segments with well-aligned, stacked disks visualized by correlative light and electron microscopy (CLEM; Eberle et al., 2012). Additionally, OS-like structures were also observed when primary rods were grafted into a late-stage retinal degeneration mouse model of autosomal dominant RP (P347S mouse; Li et al., 1996) with a virtually complete loss of photoreceptors. Interestingly, the cells remained in the subretinal space in clusters but still generated OS with similar morphological features (Eberle et al., 2012).

Gust and Reh (2011) reported that adult photoreceptors were still integration-competent, however, with significantly reduced integration potential in adult wild-type recipients. Interestingly, they also showed a strongly reduced survival potential of mature photoreceptors compared to immature ones *in vitro* which might be one factor for their low integration rates. The low survival rate in turn might be a cell dissociation effect since protocols based on enzymatic and mechanical dissociation lead to a major loss of outer segments and axonal terminals resulting in accelerated cell death (Zayas-Santiago and Derwent, 2009; Gust and Reh, 2011). It also remains to be shown whether transplanted adult rods are able to integrate into mouse models of photoreceptor degeneration.

Interestingly, survival of donor photoreceptors can be improved using anti-apoptotic factors such as X-linked inhibitor of apoptosis protein (XIAP). Treatment of P4 donor photoreceptors using adeno-associated virus (AAV) vectors expressing XIAP significantly improved survival of transplanted photoreceptors in long-term experiments (Yao et al., 2011a). XIAP-treated grafts had a higher survival rate of integrated rods up to 8 months in rd9 mice, a X-linked retinal degeneration model. Despite their improved survival, alleged integration rates varied depending on the host's age: elderly hosts were more permissive for potential cell integration when compared to younger counterparts, reflecting the influence of the host environment on transplantation outcome (Yao et al., 2011a). These results contradict previous findings using grafted neural progenitors into the mouse eye where younger hosts provided better support than older hosts for a successful transplantation outcome (Sakaguchi et al., 2003). Indeed, distinct host environments as it is also observed in diverse retinal degeneration models have been shown to have significant influence on photoreceptor transplantation success (Barber et al., 2013; see below). Nonetheless, improved donor survival using XIAP treatment may represent an interesting tool for adult donor photoreceptors to improve survival after transplantation.

## Donor photoreceptor enrichment

A major limitation to achieve significant functional improvement of photoreceptor-mediated vision might be the low number of integrating donor cells. To increase the total number of integrated cells two approaches were followed: first, manipulation of the donor cells/transplantation technology and, second, modification of the recipient retinas.

**Figure 2:**
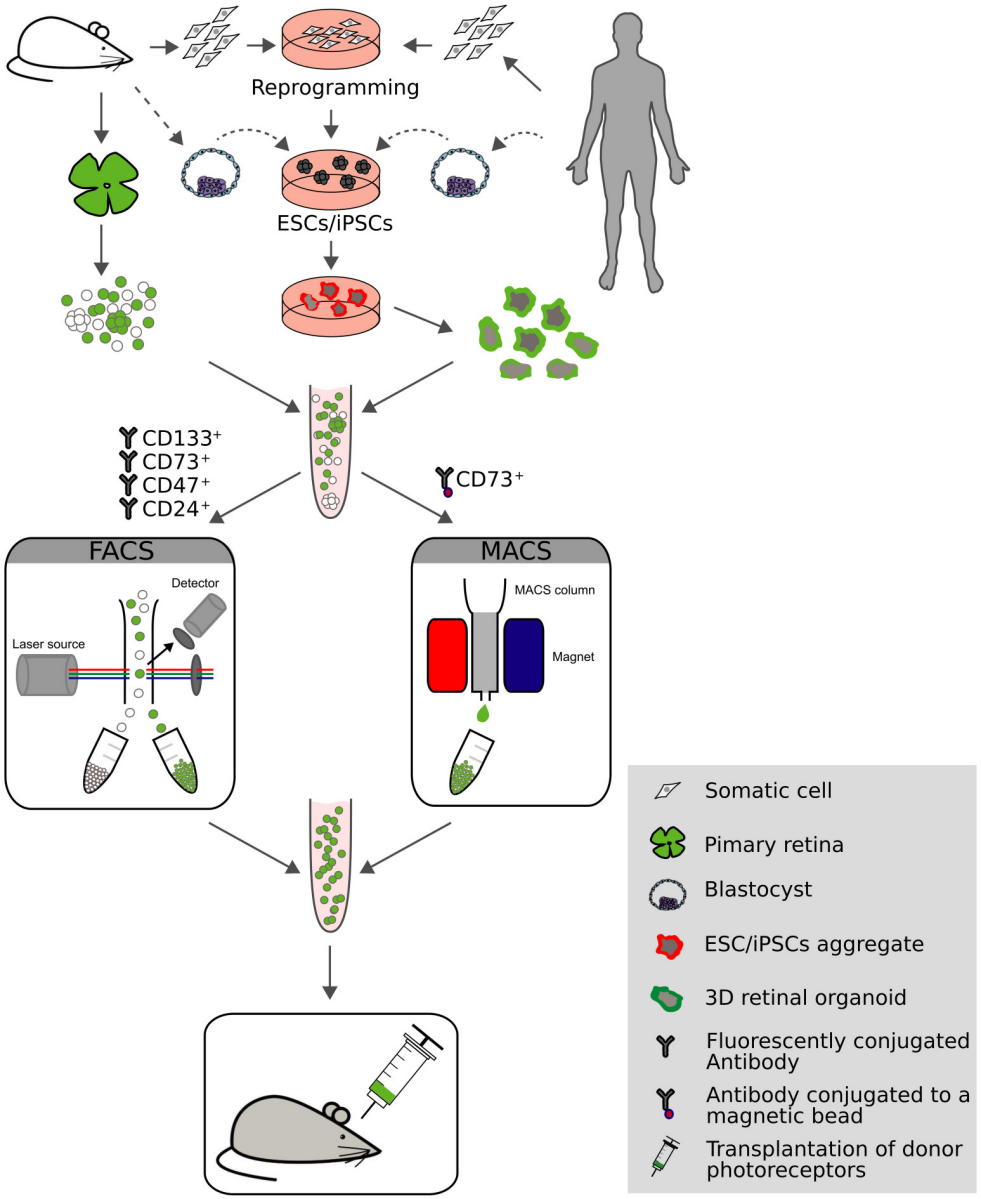
**Overview of current approaches for photoreceptor generation, sorting and transplantation**. Donor photoreceptors can be acquired from multiple sources: primary rodent retinas, embryonic stem cells (ESCs) or reprogrammed somatic cells which give rise to induced pluripotent stem cells (iPSCs). Expandable cell sources can be cultured using 3D technology resulting in the generation of photoreceptor rich retinal organoids. Donor cells can be enriched by fluorescent activated cell sorting (FACS) or magnetic activated cell sorting (MACS) using fluorescent labels or cell surface markers (CD133^+^/CD73^+^/CD47^+^/CD24^+^, or CD73^+^, respectively). Following transplantation into mouse models of retinal degeneration the potential for retinal repair is evaluated.

To increase the photoreceptor content in suspensions for transplantation, donor cells were enriched from whole retina extracts using either FACS or magnetic activated cell sorting (MACS; Figure 2). Indeed, by using transgenic mice with rod-specific expression of GFP, donor photoreceptors could be efficiently enriched to >95% by flow cytometry that resulted in significantly higher numbers of integrated photoreceptors following transplantation (MacLaren et al., 2006; Lakowski et al., 2011; Pearson et al., 2012; Barber et al., 2013; Warre-Cornish et al., 2014). Alternatively, the use of cell surface markers was exploited for photoreceptor enrichment, which presents a viable alternative for clinical application by circumventing the need for fluorescence reporter expression in donor cells, particularly when applying MACS enrichment technology, as it is easier to adapt to GMP conditions than flow cytometry. CD73 (ecto-5′-nucleotidase) was identified as a marker for rod photoreceptors (Koso et al., 2009; Eberle et al., 2011; Lakowski et al., 2011) and was used either alone (Eberle et al., 2011) or in combination with CD24 (Lakowski et al., 2011) for the enrichment of rod photoreceptors prior transplantation resulting in significant increase of integrated rods in the adult mouse retina. A similar approach was undertaken using mouse embryonic stem cell-(mESC) derived rods (see below), where a panel of antibodies was identified (CD73^+^/CD133^+^/CD24^+^/CD47^+^/CD15^−^, named photoreceptor precursor (PPr) panel*)* that exclusively label transplantation competent rods. PPr-positive rods were able to integrate to a higher extend when compared to eGFP labeled rods enriched by FACS or donor cells enriched with a single cell surface marker (Figure 2; Lakowski et al., 2015). Further, additional cell surface markers have been identified such as Cacna2d4, Kcnv2, and Cnga1 that potentially allow enrichment of rod photoreceptors but the lack of reliable antibodies currently limits their use (Postel et al., 2013; Kaewkhaw et al., 2015).

## Trans-vitreal and trans-scleral injections

**Figure 3:**
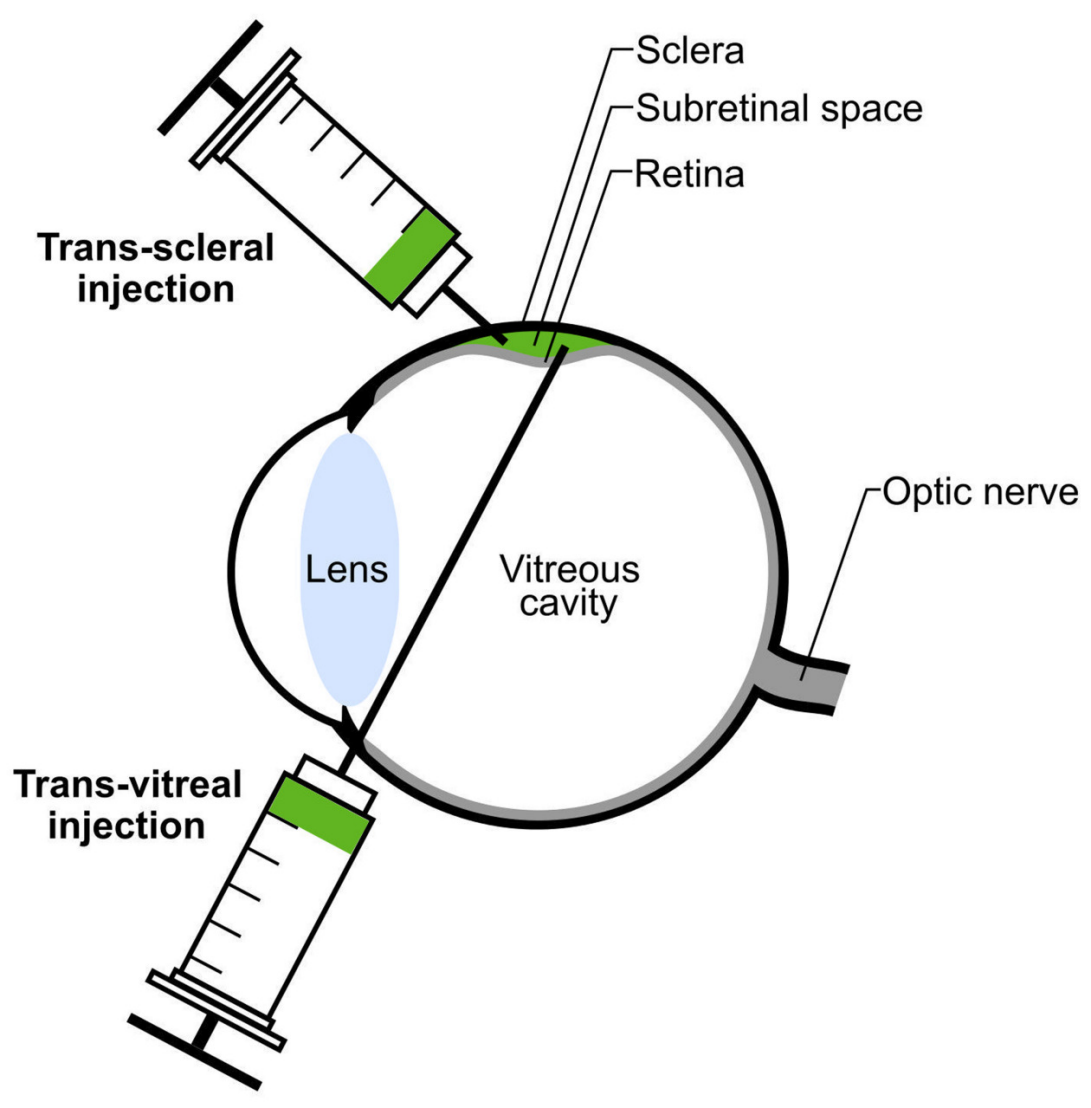
**Subretinal transplantation via trans-scleral or trans-vitreal injection**. Donor photoreceptors (green) can be transplanted into the donor's subretinal space either by trans-scleral or trans-vitreal injection. Subretinal injection leads to transient detachment of the host retina (green bulge).

The delivering technique of donor photoreceptors might impact graft efficiencies as well. Two routes are currently used for subretinal transplantation in rodents: trans-vitreal and trans-scleral injections (Figure 3). Both techniques reveal high levels of efficacy in delivering donor cells to the subretinal space but they might induce different responses in the host eye. In a trans-vitreal approach, the injection needle has to pierce through the retina, which will induce some local retinal gliosis, while a trans-scleral approach partially avoids such detrimental outcome. In addition, trans-scleral injections allow the targeting of two injection sites in one session of transplantation while such approach is difficult to be performed by a trans-vitreal route. Unfortunately, in trans-scleral injections the experimenter cannot clearly visualize the blood vessel network adjacent to the retina (choroid), thus increasing the risk of severe hemorrhage in case of damage. Conversely, trans-vitreal injections are currently used in clinical applications for cell- and gene-based therapies and thus more closely reflect a potential future clinical situation.

## Immune response following photoreceptor transplantation

Graft survival is essential for a successful cell replacement therapy, however, some studies suggest that the total number of integrated rods dramatically declines from 3 to 4 months after transplantation (West et al., 2010; Yao et al., 2011a). Immune responses are discussed as a potential factor that contributes to the poor long-term survival. The infiltration of immune relevant cells such as macrophages and T-cells into the subretinal space, as well as migration of microglia to the graft, have been observed and correlated with reduced numbers of integrated cells in the host ONL (West et al., 2010). The study by West and colleagues were performed using donor cells and recipients with partially matched haplotypes besides injection of unsorted retinal cells, that were not enriched for photoreceptors, raising the question to what extend matching haplotypes and specific cell types influence immune responses in the retina. Interestingly, systemic immune suppression by cyclosporine A resulted in improved transplantation outcome (West et al., 2010). Other immunosuppressants such as prednilosone or indomethacin have been used to reduce immune response following Müller Glia transplantation into RCS rats and allowed higher integration rates (Singhal et al., 2008).

However, the role of innate immune cells in context with retinal transplantation and degenerative disorders is not understood in a detailed manner. Recently, Neves et al. (2016) found that following photoreceptor transplantation the modulation of innate retinal immune cells via the mesencephalic astrocyte-derived neurotrophic factor (MANF) promoted retinal repair. However, the exact mechanisms that might affect the transplanted photoreceptors are not fully known. In that regard, several questions should be addressed: What is the origin and dynamics of the immune cells present in the graft? Are they resident or circulating cells? What is their contribution to graft depletion? Do different retinal degenerative diseases also lead to distinct activation patterns of immune responses? In this context a recent study using RPE transplantation provided evidence for the importance of HLA matching also in the retina: transplantations of haplotype matched iPSC-derived RPE cells into the subretinal space of monkeys lacked T-cell mediated immune responses whereas in non-matched conditions rejection processes were observed (Sugita et al., 2016). Nevertheless, it is currently under investigation to what extend graft purity and matching haplotypes contribute to immune responses and photoreceptor transplantation success.

## Manipulation of the host retina

Within the recipient retina three components were identified that might represent a major barrier for donor photoreceptor migration into the host tissue: (i) the outer limiting membrane (OLM); (ii) reactive gliosis; and (iii) the extracellular matrix (ECM).

The OLM is composed of tight junctions between rod inner segments and Müller glia (MG) end feet and constitutes a natural barrier between the ONL and the inter-photoreceptor matrix, i.e., the sub-retinal space. To compromise Müller glia cell integrity, the OLM was disrupted by: (1) siRNAs against components of the OLM, i.e., Crb1and ZO-1; or (2) the glial toxin *alpha-aminoadipic acid* (AAA). This resulted in a higher number of integrated donor cells within the host tissue (Pearson et al., 2010; West et al., 2010; Barber et al., 2013).

Upon a retinal insult, whether genetic, chemical or mechanical, Müller glia (MG) react in a non-specific manner called reactive gliosis, that is characterized by up-regulation of specific markers, hypertrophy, and sometimes proliferation. The function of gliosis has not been fully understood as it might have both beneficial and detrimental effects. In the case of retinal degenerations, the loss of photoreceptors leads to a rearrangement of the ONL and respective OLM. MG become reactive which can be identified by the presence of glial acidic fibrillary protein (GFAP) and other intermediate filaments like vimentin. The degree of MG activation is highly variable and disease specific (Hippert et al., 2015). Gliosis might increase the physical barrier of the OLM and therefore hinder the migration of transplanted photoreceptors into the host retina and formation of synaptic contacts to the second order neurons. Indeed, mouse models of retinal degeneration with strong MG activation are less permissive for donor photoreceptor integration than models with lower reactive gliosis (Barber et al., 2013). Furthermore, transplantation of retinal progenitors into the subretinal space of a mouse model lacking GFAP and vimentin lead to higher levels of donor cell migration into the retinal tissue (Kinouchi et al., 2003), suggesting that Müller glia activation might impair donor cell integration. It is hypothesized that the absence of GFAP leads to a loosening of the OLM's tight junctions and, thus, promoting cell integration. However, GFAP is also present in astrocytes which were never addressed in this context, as they reside histologically distant from the subretinal space in the GCL. Moreover, MG hypertrophy, MG swelling, and thickening of the OLM might hinder photoreceptor integration as well. Collectively, current data suggests that reactive Müller glia is detrimental for successful migration and integration of donor cells into degenerated retinas. However, further in depth studies investigating the underlying mechanism(s) will be required to develop distinct manipulations to improve the transplantation outcome.

Another target within the host tissue that might influence transplantation success in the retina are components of the ECM, since these might pose a barrier for transplanted photoreceptors to integrate and establish synaptic connections to downstream neurons. Hence, the digestion of the ECM using matrix metalloproteases (MMP2) or bacterial enzymes (such as chondroitinase ABC) might facilitate migration and synaptogenesis (Suzuki et al., 2007; Yao et al., 2011b; Barber et al., 2013).

Overall, though a number of studies have assessed in detail the characteristics of donor cells for retinal transplantation, only few reports have analyzed the host response upon photoreceptor transplantation. Thus, a systematic approach for identifying conditions within the host tissue that allow successful migration and integration of donor photoreceptors will be highly important for the development of cell-based retinal replacement therapies.

## Rod photoreceptor transplantation into retinal degeneration mouse models

The ultimate goal of photoreceptor transplantation is to repair lost visual function. Due to the availability of a wide range of transgenic and chemical degeneration and reporter strains, mice represent the main experimental species in retinal transplantation research. However, mice are nocturnal animals and do not rely as much on vision as humans, thus, some researchers estimate the normal sight of mice is on a level that would allow for a human to be recognized as legally blind (Baker, 2013). Therefore, the development of sensitive and reliable methods for the evaluation of vision improvement following cell transplantation is of utmost importance.

Indeed, improved visual function following cell transplantation using a standard test for retinal function, the electroretinogram (ERG), has only achieved very limited results. This might be explained by low numbers of functionally connected donor photoreceptors within recipient retinas as the ERG represents a bulk analysis method that summarizes electrical potentials from the whole retina (Peachey and Ball, 2003).

However, Pearson and colleagues observed some functional restoration of rod-mediated vision following rod transplantation in a mouse model of stationary night blindness using several other vision-based tests (Pearson et al., 2012). The guanine nucleotide-binding protein G subunit alpha-1 knockout mouse model (Gnat1^−/−^) shows impaired rod phototransduction and thus night blindness. Rod photoreceptors degenerate slowly in Gnat1^−/−^ mice and the authors hypothesized that at the time of transplantation the remaining endogenous photoreceptors might provide a natural scaffold for donor photoreceptors to integrate. Restoration of rod vision by photoreceptor transplantation was validated at the cellular, retinal, visual cortex as well as behavioral level using a number of methodologies including single cell electrophysiology, optokinetic tracking, cortical imaging, and a water maze behavioral test (Pearson et al., 2012; Barber et al., 2013), providing strong evidence for the feasibility of such therapeutic approach.

**Figure 4:**
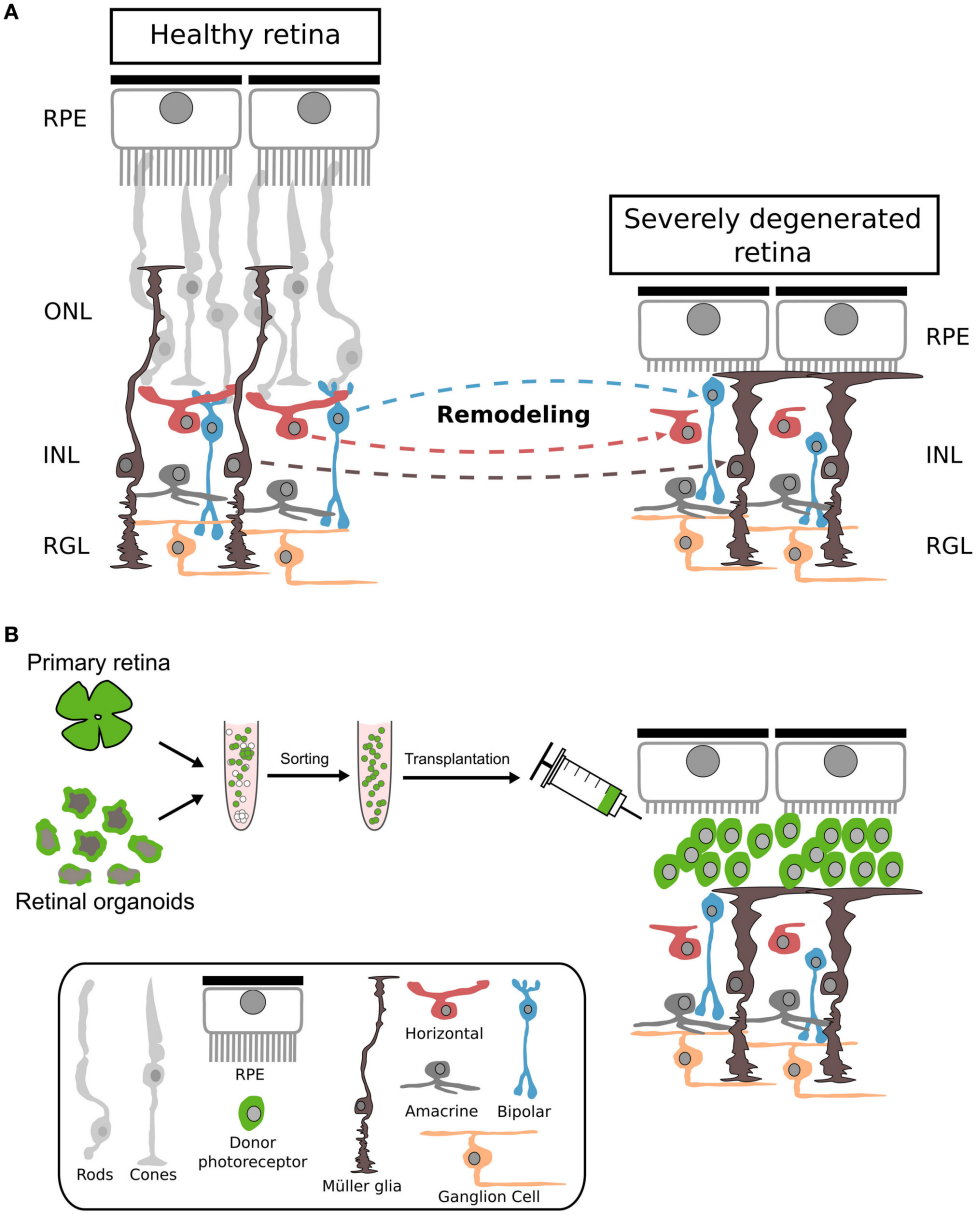
**Cellular changes during severe photoreceptor degeneration and subsequent photoreceptor transplantation. (A)** Morphological changes of different retinal cell types following complete degeneration of photoreceptors. During photoreceptor degeneration, Müller glia becomes reactive, resulting in a thickening of the OLM accompanied by reduction of dendritic arborization of bipolar and horizontal cells. Interneurons become misplaced at later stages of degeneration. RPE: retinal pigment epithelium; ONL: outer nuclear layer; INL: inner nuclear layer; RGL: retinal ganglion cell layer. **(B)** Primary or h/m ESC/iPSC-derived photoreceptor cells transplanted into a retina with complete photoreceptor degeneration remain in the subretinal space. Nrl: neural leucine zippe; eGFP: enhanced green fluorescent protein; h: human; m: mouse; ESC: embryonic stem cells; iPSC: induced pluripotent stem cells.

Actually, most transplantation studies used animal models of retinal degeneration with some remaining endogenous photoreceptors that might facilitate donor cell integration and polarization. However, end-stage degeneration with complete loss of photoreceptors might represent the first target for future clinical trials (Figures 1, 4). Therefore, mouse models with severe retinal degeneration might represent an important and more realistic environment for assessing the potential of photoreceptor replacement approaches.

Initial experiments were performed by transplanting photoreceptors into mouse models of severe retinal degeneration like the rd1 or P347S mouse lines (Eberle et al., 2012; Singh et al., 2013). Both studies provided evidence for survival of donor rods with no migration into the remaining retinal tissue. Instead, donor rods formed cell clusters within the host sub-retinal space in close contact to the recipient's INL. Interestingly, donor rods expressed phototransduction cascade as well as synaptic markers and generated disc-filled outer segment-like structures with light dependent translocation of rod transducin between cell body and outer segments, suggesting the differentiation of donor cells into mature photoreceptors (Peachey and Ball, 2003; Eberle et al., 2012). Furthermore, several vision-based tests were used, including electroretinography, light/dark box, pupillary reflex, and laser speckle cortical imaging, that suggested functional repair upon cell replacement therapy (Singh et al., 2013). Interestingly, the visual improvements were observed following rod photoreceptor transplantation into the widely used rd1 retinal degeneration mouse model which is characterized by a rapid and severe photoreceptor loss. However, recent publications identified a second mutation in rd1 mice on the Gpr179 gene that effects ON-bipolar cells (Peachey et al., 2012; Balmer et al., 2013; Nishiguchi et al., 2015), the prime target of rod signaling. Only after backcrossing rd1/3H9 to a C57BL/6j background, thereby eliminating the mutation, allowed functional repair by gene therapy, thus explaining the failure of several earlier gene therapeutic attempts to repair retinal function in this model despite rescue of rod photoreceptors (Bennett et al., 1996). Recently, the function of GPR179 protein was identified and showed to be required for depolarizing ON-bipolar cells (Peachey et al., 2012). Thus, further experiments are required to determine the feasibility of cell replacement therapies in severely degenerated recipients like the rd1 mouse.

Another interesting question is, to what extent the transplantation of a photoreceptor suspension into the sub-retinal space of severely degenerated retinas allows the (self-) organization/reconstruction of a highly polarized structure as the ONL. The clusters formed by donor rods in the sub-retinal space did not show an obvious polarization or organization and frequently donor photoreceptors did not generate the characteristic stretched morphology with OSs contacting the RPE and synapses localized to the IPL (Eberle et al., 2012). However, the OSs of donor rods in rd1 mice showed a preferential orientation toward the RPE suggesting some degree of polarization within the host tissue (Singh et al., 2013).

Furthermore, it has not been analysed whether endogenous Müller glia processes contact or enwrap grafted photoreceptors as it is observed in wild-type retinas. Thus, further studies have to be conducted to assess polarization of transplanted donor photoreceptors in detail as well as determining the behavior of the surrounding host cells (Müller glia, RPE and second order neurons) following engraftment (Figure 4). Additionally, the use of scaffolds or grafting of photoreceptor sheets (Seiler and Aramant, 2012; Assawachananont et al., 2014) might represent alternative routes for the generation of proper organized photoreceptor transplants.

## Transplantation of cone/cone-like photoreceptors

Rod photoreceptors are important for vision under dim light conditions but human vision is mainly dependent on cone photoreceptors that mediate daylight and color vision besides visual acuity due to their concentration in the macula. Conversely, the mouse retina is adapted to night vision and cones correspond to only 3% of all photoreceptors. Thus, to investigate cone photoreceptor replacement therapy as it will be necessary for macular degenerative diseases (e.g., AMD) or late stage retinitis pigmentosa (RP) has been challenging due to the absence of reliable reporter lines and comprehensive donor cell sources.

The first study addressing cone transplantation relied on the use of a Crx-GFP reporter line where GFP expression is under the control of a cone-rod homeobox (Crx) promoter, labeling both cone and rod photoreceptors (Lakowski et al., 2010). Crx is a transcription factor that is expressed in all photoreceptors after retinal progenitors commit to the photoreceptor lineage. Crx-GFP positive cells were isolated at different developmental stages from E12.5 to P3, sorted by flow cytometry and transplanted into wild-type mice and a mouse model of cone degeneration. There was an increase in the number of integrated Crx-GFP^+^ photoreceptors, both rods and cones, from E12.5 to P3 but the total number of Crx-GFP^+^ cells within the ONL positive for RXR-gamma, a cone photoreceptor marker, was significantly higher when donor cells were isolated at embryonic stages. The isolation and enrichment of mixed photoreceptor populations, i.e., FAC-sorted Crx-GFP positive photoreceptors, does not dissect the competence of cones to integrate when transplanted as a pure population, whether into a rod- or cone-rich environment (Lakowski et al., 2010). Additionally, the total number of cones in an adult mouse retina is ~1.8 × 10^5^ cells (Jeon et al., 1998) which limits the possibility to study cone photoreceptor transplantation.

To circumvent this drawback, our lab took advantage of the neural retina leucine zipper transcription factor knockout (Nrl^−/−^) mouse (Mears et al., 2001) in which the absence of this rod-determining transcription factor results in a rod-less retina where rods are converted into cone-like cells expressing only short wavelength (S-) opsin (Mears et al., 2001). Cone-like photoreceptors closely resemble wild-type cones at the morphological, histological, electrophysiological and molecular level (Daniele et al., 2005; Nikonov et al., 2005). These Nrl^−/−^ mice were crossed with an ubiquitous reporter mouse line (actin-GFP) in order to trace cone-like cells after transplantation into a mouse model of cone degeneration—the cone photoreceptor function loss 1 (CPFL1) mouse (Chang et al., 2002, 2009). Here, cone-like photoreceptors integrated into the recipient ONL and resembled mature photoreceptors as they formed outer-segments and expressed photo-transduction and synaptic markers. Functional analysis was performed by micro-electrode arrays (MEA), that allow the simultaneous read out of hundreds of retinal ganglion cells (RGCs). These measurements showed spike train responses in RGCs under daylight conditions (Santos-Ferreira et al., 2015). Importantly, sham or rod photoreceptor injected CPFL1 hosts were unable to detect high-intensity light stimuli. These results suggest on the one side functionality of transplanted cone-like photoreceptors to detect daylight stimuli and on the other side proper connection of donor cells to the retinal circuitry of the recipient. Therefore, MEA measurements represent a highly sensitive tool to assess functional integration of photoreceptors following transplantation. However, MEA measurements were only performed 4 weeks after grafting despite long term survival (up to 6 months) of donor cells. Thus, it will be important to assess whether the observed functional restoration is maintained over such extended periods after transplantation.

Although cone-like cells were used as an alternative to cone photoreceptors, they are, in fact, not true cones. Therefore, several reporter lines have been developed in order to label cone photoreceptors (Fei and Hughes, 2001; Smiley et al., 2016). Smiley et al. (2016) generated a reporter line wherein GFP is under the control of the coiled-coil domain containing *136* (Ccdc136) promoter. Despite heterogeneous expression of GFP throughout more than one cell population (cones and rod bipolars), GFP-positive cones are solely labeled until P14, allowing their enrichment using FACS. Transplantation of E17.5 Ccdc136^GFP/+^ sorted cell fractions resulted in integrated photoreceptors in C57BL/6j wild-type hosts but these cells lacked the expression of cone-specific photo-transduction markers (Smiley et al., 2016) in contrast to Santos-Ferreira et al. (2015) who showed cone markers (cone arrestin, s-opsin) in reporter labeled cells following cone-like photoreceptor transplantation into CPFL1 hosts. Interestingly, in both studies the reporter labeled cells within the host ONL frequently exhibited rod-like morphologies, that might be explained by the exchange of cell material between donor and host photoreceptors (see below).

## Transplantation of donor photoreceptors derived from pluripotent stem cells

Despite all major achievements in restoring rod- and cone-mediated vision, it is clear that primary cells cannot be used in a clinical application for photoreceptor replacement purposes without generating multiple ethical and logistical concerns. Thus, an expandable, renewable source of donor cells has been pursued and pluripotent (induced or embryonic) stem cells (iPSC/ESC) represent an excellent alternative to primary cells. Several laboratories are focused on the development of differentiation protocols from iPSC/ESCs to photoreceptor fate and, nowadays, several protocols with varying efficiencies are available but the detailed discussion of these are beyond the scope of this review. We will focus on transplantation studies using human or mouse iPSC/ESC-derived photoreceptors as donor cells.

The first attempt to transplant pluripotent stem cell-derived photoreceptors was performed by Lamba et al. (2009) using human embryonic stem cell (hESC)-derived photoreceptors as donor cells. Here, hESC-derived photoreceptors were labeled with a lentiviral construct expressing eGFP under the human elongation factor 1a (hEF1a) promoter in order to trace donor cells after transplantation. Human ESC-derived photoreceptors were grafted in both the vitreal and subretinal space of mice and showed high migratory potential reaching all the nuclear layers of the retina. Importantly, donor GFP-positive cells integrated into the host's ONL and displayed a mature photoreceptor morphology as well as positivity for recoverin and synaptophysin. Besides such promising histological data also indications for functional recovery in the retinal degeneration Crx^−/−^ mouse model was reported (Lamba et al., 2009).

Also iPSC-derived photoreceptors were transplanted with varying degree of success (Lamba et al., 2010; Tucker et al., 2011; Homma et al., 2013; Figure 2). Using a genetically encoded reporter mouse iPSC reporter line (actin dsRed), dsRed^+^ iPSC-derived photoreceptor precursors were negatively-sorted for stage-specific embryonic antigen-1 (SSEA1) to eliminate potential proliferating and thus tumorigenic stem cells and then grafted into rhodopsin knock-out mice at the age of 21 days. Following transplantation fluorescently labeled cells were present in the host ONL, expressed mature photoreceptor markers and recovered some visual function as ERG measurements showed a 100 μV improvement in the b-wave (Tucker et al., 2011). Although this study provides proof-of-concept for miPSC-derived photoreceptor transplantation, a more systematic analysis would be required to dissect the potential of iPSC-derived photoreceptor for visual repair, especially at the functional level. Homma and colleagues addressed this issue by generating a mouse iPSC cell line from the Nrl-GFP mouse and transplanted iPSC-derived rods into the wild-type and dystrophic retina. Calcium imaging was performed showing that grafted photoreceptors had similar intracellular calcium oscillations upon pharmacological stimulation as endogenous rod photoreceptors (Homma et al., 2013).

The above initial studies used pluripotent stem cell-derived photoreceptors differentiated with 2D culture systems. However, culture methods were significantly improved with the ground breaking studies of the Sasai laboratory who introduced a 3D culturing system which allows mouse and hESC to self-organize and differentiate along the retinal lineage by forming organoids. These retinal organoids continue to develop into an optic cup and subsequently pseudo-stratified retinal tissue (Eiraku et al., 2011; Nakano et al., 2012). Retinal organoid technology has become a widely used method to generate transplantable photoreceptors as demonstrated by several studies (Gonzalez-Cordero et al., 2013; Assawachananont et al., 2014; Decembrini et al., 2014; Santos-Ferreira et al., 2016b). Transplantation of retinal organoid-derived photoreceptors showed similar results to grafted primary photoreceptors. Integrated photoreceptors, whether grafted into wild-type or photoreceptor degeneration mouse models, acquired a mature morphology, and expressed phototransduction and synaptic markers. However, two studies showed that integration rates of mouse ESC-derived photoreceptors were significantly lower when compared to transplanted primary photoreceptors (Gonzalez-Cordero et al., 2013; Decembrini et al., 2014).

Currently, studies assessing functional recovery after transplantation of retinal organoid-derived photoreceptors are severely limited as the cited studies mainly analyzed survival, differentiation, and maturation of donor photoreceptors. Particularly, transplantation studies using photoreceptors isolated from human PSC-derived organoids have not been published yet. However, with the generation of photoreceptor-specific human reporter ESC lines (Kaewkhaw et al., 2015), implementation of protocols for the generation of retinal organoids from hiPSCs (Zhong et al., 2014)/hESCs (Shirai et al., 2016), and given the high number of laboratories currently working on this topic several publications are awaited within the near future. Importantly, the extended time lines for the generation of cone (~60–80 days) or rod (~120–200 days) photoreceptors besides high variability in the differentiation process significantly extends the analysis and experimental set up in comparison to the mouse system.

## Paradigm shift in photoreceptor replacement therapy

**Figure 5:**
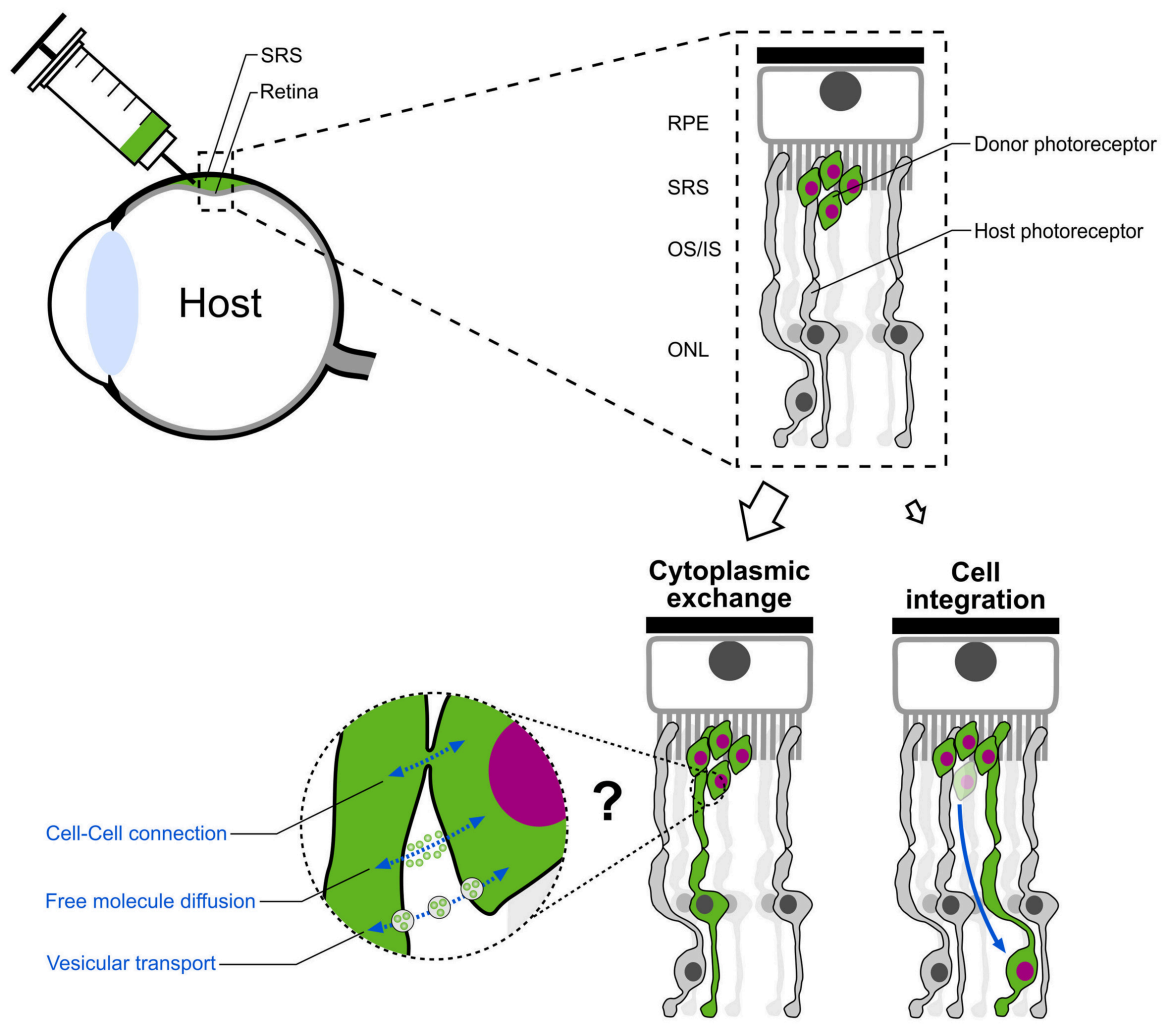
**Paradigm shift in photoreceptor replacement therapy**. Following subretinal transplantation, structural integration of donor photoreceptors was assumed as the main mechanism for vision improvement in previous pre-clinical studies. Instead, the majority of “integrated” donor cells in the ONL of the host retina represent host photoreceptors which exchanged cytoplasmic material with donor photoreceptors located in the subretinal space. Cell-cell connections, free molecule diffusion or vesicular transport are assumed as possible mechanisms. SRS, subretinal space; RPE, retinal pigment epithelium; OS/IS, outer segment/inner segment; ONL, outer nuclear layer.

The common notion that donor photoreceptors migrate and structurally integrate into the recipient photoreceptor layer was recently challenged (Pearson et al., 2016; Santos-Ferreira et al., 2016a; Singh et al., 2016). The three studies provide strong evidence, that the majority of donor photoreceptors instead remain in the sub-retinal space and exchange cytoplasmic material (including fluorescent reporter proteins) with endogenous host photoreceptors rather than structurally integrating into the recipient retina. This results in the labeling of host photoreceptors and consequently misinterpretation of labeled cells as structurally integrated donor cells (Figure 5). This material exchange is limited to components of the cytoplasm without translocation of the nucleus. Thus, a complete fusion, as it was seen between transplanted bone marrow derived progenitor cells and diverse cell-types in several tissues (Kemp et al., 2012; Yamashita et al., 2012) which resulted in the formation of bi-nucleated perikaryons, can be ruled out for the observed phenomenon in the retina. Instead, other cell-cell interactions like tunneling nanotubes or vesicular transport are hypothesized as potential transfer mechanisms.

The observed material transfer between donor and host photoreceptors has important implications for cell-based treatment approaches in retinal degeneration as it demands a reinterpretation of the above discussed former studies. Several topics have to be reconsidered including the general migratory and synaptogenic potential of donor photoreceptors. Therefore, proper animal models with severe retinal degeneration, i.e., complete photoreceptor loss, in which no material exchange between donor and host cells has been observed, should be used as benchmark models for photoreceptor replacement studies (Singh et al., 2013; Santos-Ferreira et al., 2016a).

On the other hand, the transfer of biomaterial might represent an unexpected novel way for the development of cell-based therapeutic interventions in retinopathies. Though it still remains to be shown to what extent aberrant photoreceptors also exchange cellular content with donor cells. The observed functional improvements in mouse models of rod (Pearson et al., 2012; Barber et al., 2013) and cone (Santos-Ferreira et al., 2015) degeneration after photoreceptor transplantation might point toward a cell support therapy, in which donor cells transfer “healthy” components to diseased host photoreceptors allowing functional repair.

## Concluding remarks

Significant progress has been achieved within the last decade in the evaluation and development of photoreceptor transplantation strategies aiming to treat retinal degenerative diseases. Besides the surgical accessibility of the retina, its separation from the rest of the body and partially immune privileged environment, donor photoreceptors have to generate only short processes to contact second order neurons; thus the retina might serve as a blueprint for cell replacement approaches in the central nervous system.

Young post-mitotic photoreceptor precursors rather than proliferating retinal progenitor cells were identified as the most successful cell-type for photoreceptor grafting as they showed long term survival and maturation following transplantation into the adult degenerated mouse retina (MacLaren et al., 2006; Bartsch et al., 2008) culminating in functional improvements by rod transplantation in mouse models of RP (Pearson et al., 2012) and daylight vision repair by donor cone-like photoreceptors grafted into a cone degeneration model (Santos-Ferreira et al., 2015). Additionally, technologies for the enrichment of rod photoreceptors based on photoreceptor-specific fluorescent donor lines (MacLaren et al., 2006) besides cell surface markers using flow cytometry (Lakowski et al., 2011) or MACS (Eberle et al., 2011) allowed significant improvements in transplantation outcomes.

Importantly, the observed functional improvements by photoreceptor transplantation in mouse models of retinal degeneration that still contain significant amounts of endogenous photoreceptors might be achieved by the transfer of cytoplasmic material from donor to host photoreceptors rather than structural migration and integration of grafted cells (Pearson et al., 2016; Santos-Ferreira et al., 2016a). These findings demand a reconsideration of cell-based strategies for treating retinal degenerative diseases characterized by dysfunctional but still existing photoreceptors that might benefit from therapeutic support by donor cells (cell support therapy). Conversely, retinal degeneration characterized by complete loss of photoreceptors has to be considered as the main target for photoreceptor replacement approaches (cell replacement therapy).

Another essential step toward the development of cell-based therapies in the retina represents the ground-breaking technology of PSC-derived retinal organoids and their use for the generation of high amounts of transplantation-competent photoreceptors (Eiraku et al., 2011; Nakano et al., 2012; Gonzalez-Cordero et al., 2013; Decembrini et al., 2014; Santos-Ferreira et al., 2016b; Völkner et al., 2016). However, given the high variability still observed in the differentiation outcome of human PSCs further detailed knowledge of retina and photoreceptor development for targeted modification of molecular pathways will be essential for developing pipelines that allow clinical grade photoreceptor transplant production according to good manufacturing practice (GMP) guidelines (Wiley et al., 2016). Furthermore, the extended time periods for the generation of human photoreceptors, particularly rods, with more than 100 days *in vitro*, represents a major technical challenge for robust clinical grade cell production.

As retina organoids show significant heterogeneity the enrichment of target cells by GMP adaptable techniques will be essential for future clinical use. MACS might represent a potential method for such purification as it has been established for cell sorting approaches in clinical applications, e.g., hematopoietic cell-therapies. However, although potential cell surface markers for cell sorting have been identified for rods (Eberle et al., 2011; Lakowski et al., 2011; Kaewkhaw et al., 2015), specific markers or marker-panels for human cones still need to be established.

The observation of cell material transfer between donor and host photoreceptors has particular implications on our view of connectivity and synapse formation between donor cells and second order neurons, as most of the previously described connections might actually represent still existing synapses between endogenous photoreceptors and their target cells rather than newly formed interactions. Therefore, detailed studies have to assess the connections of donor photoreceptors to endogenous bipolars and horizontals. Particularly when considering the significant remodeling and dendritic arborization in late stage retinal degeneration, where most, if not all, photoreceptors have been lost, the generation of new specific and proper synaptic connections might represent one of the major challenges in the field.

Finally, in the long run one of the major disease conditions to be targeted by retinal cell transplantation is late stage dry AMD with its deleterious loss of both, cone photoreceptors and RPE, that might demand co-injection strategies. With PSC-derived RPE transplantation already performed in first trials (Schwartz et al., 2015), a blueprint to bring stem cell-based cell products to the clinic has been introduced, which will be of enormous help for also introducing photoreceptor transplantation to patients. However, animal models that can serve as appropriate models for late stage dry AMD are missing and their development would be of tremendous help to pre-clinically study cone and RPE replacement approaches. Hence, photoreceptor transplantation represents a highly dynamic research field that considerably progressed within the last decade, but with a number of potential road blocks still remaining to be tackled on the path to clinical application.

## Author contributions

TS, OB, and MA wrote the manuscript. TS and OB made the figures. MA provided funding and supervised the whole review.

### Conflict of interest statement

The authors declare that the research was conducted in the absence of any commercial or financial relationships that could be construed as a potential conflict of interest. The handling Editor declared a shared affiliation, though no other collaboration, with the authors and states that the process nevertheless met the standards of a fair and objective review.
